# Exploring the differences in ICD and hospital morbidity data collection features across countries: an international survey

**DOI:** 10.1186/s12913-021-06302-w

**Published:** 2021-04-07

**Authors:** Lucia Otero Varela, Chelsea Doktorchik, Natalie Wiebe, Hude Quan, Catherine Eastwood

**Affiliations:** 1 University of Calgary Cumming School of Medicine, TRW 5th Floor, 3280 Hospital Drive NW, Calgary, Alberta T2N 4N1 Canada; 2 Libin Cardiovascular Institute of Alberta, HMRB (Room 72), 3310 Hospital Drive NW, Calgary, Alberta T2N 4N1 Canada

**Keywords:** International classification of diseases, Surveys and questionnaires, Data collection features, Hospital morbidity database, International comparability

## Abstract

**Background:**

The International Classification of Diseases (ICD) is the reference standard for reporting diseases and health conditions globally. Variations in ICD use and data collection across countries can hinder meaningful comparisons of morbidity data. Thus, we aimed to characterize ICD and hospital morbidity data collection features worldwide.

**Methods:**

An online questionnaire was created to poll the World Health Organization (WHO) member countries that were using ICD. The survey included questions focused on ICD meta-features and hospital data collection systems, and was distributed via SurveyMonkey using purposive and snowball sampling. Accordingly, senior representatives from organizations specialized in the topic, such as WHO Collaborating Centers, and other experts in ICD coding were invited to fill out the survey and forward the questionnaire to their peers. Answers were collated by country, analyzed, and presented in a narrative form with descriptive analysis.

**Results:**

Responses from 47 participants were collected, representing 26 different countries using ICD. Results indicated worldwide disparities in the ICD meta-features regarding the maximum allowable coding fields for diagnosis, the definition of main condition, and the mandatory type of data fields in the hospital morbidity database. Accordingly, the most frequently reported answers were “reason for admission” as main condition definition (*n* = 14), having 31 or more diagnostic fields available (*n* = 12), and “Diagnoses” (*n* = 26) and “Patient demographics” (*n* = 25) for mandatory data fields. Discrepancies in data collection systems occurred between but also within countries, thereby revealing a lack of standardization both at the international and national level. Additionally, some countries reported specific data collection features, including the use or misuse of ICD coding, the national standards for coding or lack thereof, and the electronic abstracting systems utilized in hospitals.

**Conclusions:**

Harmonizing ICD coding standards/guidelines should be a common goal to enhance international comparisons of health data. The current international status of ICD data collection highlights the need for the promotion of ICD and the adoption of the newest version, ICD-11. Furthermore, it will encourage further research on how to improve and standardize ICD coding.

**Supplementary Information:**

The online version contains supplementary material available at 10.1186/s12913-021-06302-w.

## Background

The International Classification of Diseases (ICD) is the reference standard for reporting diseases and health conditions, and is the foundation for the identification and comparison of health trends and statistics globally [1]. Using this diagnostic classification, data generated at every patient encounter with the healthcare system are abstracted and coded into administrative health databases. Administrative health data (and particularly hospital morbidity data) are generally referred to as ICD-coded data, which contain rich clinical and health services information. These data have been increasingly and widely used for disease surveillance, research, decision- and policy-making, resource allocation, as well as quality and safety, to ultimately improve population health [2].

Since ICD was first created in 1983, revisions have continued on an approximately decade-to-decade basis, resulting in different versions, developed by the World Health Organization (WHO). Currently, the 10th version (ICD-10) is the most frequently used in the world, although its adoption and utilization vary from country to country, and national clinical modifications have been made to address country-specific needs (e.g., Australia ICD-10-AM, USA ICD-10-CM, Canada ICD-10-CA, Germany ICD-10-GM or Korea ICD-10-KM) [3, 4]. Despite the attempts by WHO to regulate these amendments, a number of significant differences have arisen. For example, some countries have added more specific clinical coding options, resulting in a greater number of codes compared with the original version, while others have modified index terms to simplify ICD-10 [5].

The development of clinical modifications has raised concerns about the impact on comparability of administrative health data, as heterogeneity of data can hinder comparisons and limit generalizability of observed findings [6]. For this reason, the WHO has released a scientifically and technologically updated version, the 11th version for Mortality and Morbidity Statistics (ICD-11 MMS), with improved usability and comprehensiveness [1, 7]. Accordingly, with the overarching goal of facilitating and optimizing the implementation of ICD-11 worldwide, the purpose of this international survey was to establish the current status of ICD use for coding morbidity statistics and to characterize hospital morbidity data collection worldwide, with a focus on ICD meta-features (i.e., number of allowable diagnosis fields, preferred definition of main condition and the reporting of diagnosis timing).

## Methods

### Participants, sampling frame and procedure

This descriptive survey was aimed to poll the World Health Organization member countries that were using ICD. Purposive sampling was used to select participants who had a well-informed understanding of ICD and the data collection system in their country. These individuals held one of the following positions: health information managers, representatives from the Ministry of Health, representatives from a national organization that maintains or disseminates hospital data, educators of coding specialists, and persons involved with hospital data collection.

To reach a wider audience, snowball sampling was also used, i.e., we found new participants based on referral from initial sample participants [8]. To this effect, we contacted representatives from international organizations specialized in the topic and asked them to complete and distribute the survey. These organizations include the WHO Family of International Classifications (WHO-FIC) Collaborating Centres, the Pan American Health Organization (PAHO), the International Federation of Health Information Management Association (IFHIMA) and RELACSIS (Red Latinoamericana y del Caribe para el fortalecimiento de los Sistemas de Salud, *transl.* The Latin American and Caribbean Network for the enhancement of Health Systems) [9–12].

### Survey design

Survey questions and possible answers were derived from an in-depth review of the peer-reviewed and grey literature, as well as four focus groups held with experts in ICD coding and administrative health data. First, we performed a Google search to understand how ICD was used and collected worldwide. Second, we searched the websites of organizations specialized in the topic, such as WHO-FIC Collaborating Centres, or the Organisation for Economic Co-operation and Development (OECD) [9, 13]. Lastly, questions and answers (e.g., cutoffs for the number of coding fields, mandatory data fields) were formulated from peer-reviewed literature and experts’ opinions, and were finalized over the course of four focus groups. The survey was thus co-developed with experts in ICD coding and administrative data to address our specific research objectives and ensure the presence of face validity. Furthermore, to increase outreach and minimize language barriers, the questionnaire was translated into Spanish. Therefore, two versions were available for participants to choose from (English and Spanish).

### Survey content

Questions included methods for and features of hospital morbidity data collection (e.g., number of intervention coding fields, mandatory data fields, electronic abstracting system) and specifically, ICD meta-features (i.e., number of allowable diagnosis, preferred definition of main condition and the reporting of diagnosis timing). Within coding practices, the main condition is a mandatory field and can be defined in two ways. In situations where there is more than one condition, the main condition is typically defined as: 1) the “reason for admission”, or 2) the most responsible diagnosis for the patient’s hospital stay or “resource use”, as documented at the end of the episode of health care. “Diagnosis timing” was also explored for its contribution to surveillance of healthcare related adverse events. “Diagnosis timing” requires the coding personnel to enter information on whether the patient’s diagnosis was present on admission or developed after admission.

The questionnaire consisted of closed-ended questions where the participants could choose an answer from a set of options. Some questions led to a subsequent open-ended question if selecting “No” or “Other”, for the purpose of gaining further clarification. For instance, if the participant’s country did not have national standards for data collection using ICD codes, the next question would allow the participant to explain what type of standards their country possessed (e.g., local, regional). Open-ended responses applied to questions n° 3, n° 7, and n° 11. Survey respondents shared additional information about important features in their country’s data collection system using a free-text box (question n°10). The questions comprising the survey are listed in Additional file 1.

### Data collection

The questionnaire was administered by SurveyMonkey® (www.surveymonkey.com, Palo Alto, California, USA), and was delivered to respondents both electronically and in person. Potential participants were contacted initially with an individualized email describing the study, its purposes, the inclusion criteria and the survey link. We also asked participants to forward the email to colleagues (from their or another country) who held one of the desired roles and could answer the questions about ICD coding. Two reminders were sent 3 and 6 months after the initial email. We also contacted more organizations and individuals through a second round of emails, with efforts to increase participation of countries from the continents that were under-represented. Additionally, paper cards promoting the study and the survey were designed, and were handed out to delegates by team members at the 2017 and 2018 WHO-FIC annual meetings. The survey was available from September 2017 until December 2018.

### Data analysis

Once data collection was completed, survey answers were first tabulated and grouped by country to better assess the differences between them. Therefore, responses provided by participants from the same country were collated/cross-checked, and when they differed, all answers were reported for further discussion.

Categorical data from closed questions was analyzed using descriptive statistics (i.e., percentages). Qualitative data from open-ended questions was first translated into the English language, then reviewed and analyzed by three of the research team members (LOV, CD, NW). They agreed upon the classification of information based on the questions (e.g., maximum number coding fields, mandatory data fields, definition of main condition) and also, based on the themes that arose during analysis (e.g., country-specific characteristics). Differences between countries in the aforementioned topics were explored and are presented in table and narrative form with descriptive analysis.

## Results

### Respondent characteristics

Overall, there were 47 participants who responded to our survey. Some of these respondents were from the same country, resulting in a total of 26 participating countries. The completion rate was 47/54 (87%), as 7 respondents started but did not complete the survey. A response rate was not calculated as we used snowball sampling. We contacted countries through the WHO and their Collaborating Centres, and while there are 117 countries who currently use ICD [1], we were only able to reach 26 of them. However, we achieved representation of countries from all continents, whose geographic distribution was: Europe (27.6%); North America (21.3%); Asia (17%); South America (14.9%); Africa (12.8%); and Oceania (6.4%). Specific countries and frequencies of respondents are listed in Table 1.

**Table 1:** ICD data collection features

Country (by continent)	N° of respondents	Definition of “main condition”	Maximum allowable coding fields for	
			Diagnoses	Hospital interventions
**Africa**				
Botswana	1	Reason for admission	31 or more (limited)	No
Mauritius	1	Reason for admission	1–6	1–6
Nigeria	3	Reason for admission	16–30 31 or more (limited) Unlimited	1–6 31 or more (limited)
Tanzania	1	Reason for admission	Unlimited	Unlimited
**America (North)**				
Barbados	2	Resource use	I don’t know	I don’t know
Canada	1	Resource use	16–30	16–30
Jamaica	2	Reason for admission	7–15 31 or more (limited)	7–15 31 or more (limited)
United States of America	5	Reason for admission	16–30	1–6 16–30 Unlimited
**America (South)**				
Chile	2	Resource use	7–15	7–15
Guatemala	3	Resource use	1–6	1–6
Paraguay	1	Resource use	I don’t know	I don’t know
Uruguay	1	Reason for admission	1–6	1–6
**Asia**				
India	1	Reason for admission	1–6	No
Indonesia	3	Resource use	1–6 16–30	1–6 16–30
Iran	1	Reason for admission	Unlimited	Unlimited
Saudi Arabia	1	Reason for admission	16–30	16–30
South Korea	1	Resource use	31 or more (limited)	31 or more (limited)
Thailand	1	Reason for admission	Unlimited	Unlimited
**Europe**				
Germany	1	I don’t know	I don’t know	I don’t know
Italy	2	Resource use	–	–
Netherlands	2	Both^a^	Unlimited	Unlimited
Spain	1	Resource use	16–30	16–30
Sweden	3	Both^a^	7–15 31 or more (limited) Unlimited	7–15 31 or more (limited) Unlimited
United Kingdom	4	Resource use	16–30 31 or more (limited) Unlimited	16–30 31 or more (limited) Unlimited
**Oceania**				
Australia	2	Reason for admission	31 or more (limited)	31 or more (limited)
New Zealand	1	Reason for admission	31 or more (limited)	31 or more (limited)
	47			

Non-response was represented with a dash (−)

^a^Respondents from that country reported both answers “resource use” and “reason for admission”

Between-country differences in morbidity data collected were assessed. Generally, these differences were grouped into four distinct categories: main condition, maximum coding fields, mandatory data fields, and country-specific characteristics.

### ICD and hospital morbidity data collection features

#### Main condition

Variation in ICD data collection features is presented in Table 1. Fourteen of the 26 respondent countries reported using “reason for admission” as the main condition definition (53.8%). However, 9 of the 26 countries stated that the diagnosis that led to a prolonged length of stay, or that occupied the greatest resources (i.e., “resource use”) was the primary definition for main condition (34.6%). Responses differed between respondents in two countries (Netherlands and Sweden), therefore both options were reported as the defining variables for the main condition definition. Only one country in Europe reported not knowing. Despite the within-country and within-continent differences, participating countries from Africa and Oceania were consistent in their responses.

#### Maximum coding fields

Countries also varied in data collection norms on the maximum amount of allowable coding fields for both diagnoses and hospital procedures/interventions. Twenty-two countries selected a numerical response for diagnosis coding fields (i.e., all responses except for “I don’t know” and one non-response). The majority of countries (*n* = 12) reported either having 31 or more (limited) coding fields (*n* = 6) or unlimited coding fields for diagnoses (*n* = 6). The remaining countries reported having 16–30 coding fields (*n* = 5) or ranged from 1 to 15 diagnosis coding fields (*n* = 5). In regards to the hospital interventions, most of the countries reported the same maximum number of coding fields for both diagnosis and procedures, with the exception of Botswana and India, as interventions are not coded in their hospital morbidity database.

#### Mandatory data fields

The type of information that is required to be abstracted from the medical charts about the patient’s stay (i.e., mandatory data fields) also varied from country to country (see Table 2). All 26 countries reported using “Diagnoses” (*n* = 26), closely followed by “Patient demographics” (*n* = 25). Conversely, 11 out of the 26 countries did not report collecting information pertaining to “Diagnosis timing” (42.3%), while nine did not require collecting “Physician information” (i.e., specialty) (34.6%). Thus, the latter were the two least frequently required data fields.

**Table 2:** Mandatory data fields collected in the hospital morbidity database

Country (by continent)	Patient demographics	Admission type^a^	Discharge disposition^b^	Admission unit	Physician information^c^	Diagnoses	Diagnosis timing^d^
**Africa**							
Botswana	✔	✔	✔	✔	✔	✔	
Mauritius	✔	✔	✔	✔	✔	✔	
Nigeria	✔		✔	✔	✔	✔	✔
Tanzania	✔	✔	✔	✔	✔	✔	
**America (North)**							
Barbados	✔	✔	✔	✔	✔	✔	✔
Canada	✔	✔	✔	✔	✔	✔	
Jamaica	✔		✔	✔	✔	✔	✔
USA	✔	✔	✔	✔	✔	✔	✔
**America (South)**							
Chile	✔		✔	✔	✔	✔	
Guatemala	✔	✔		✔		✔	
Paraguay	✔	✔	✔			✔	
Uruguay	✔		✔			✔	
**Asia**							
India						✔	
Indonesia	✔	✔	✔	✔	✔	✔	✔
Iran	✔	✔	✔	✔	✔	✔	✔
Saudi Arabia	✔	✔	✔	✔	✔	✔	✔
South Korea	✔	✔	✔	✔	✔	✔	✔
Thailand	✔		✔	✔	✔	✔	✔
**Europe**							
Germany	✔	✔	✔	✔		✔	
Italy	✔	✔	✔			✔	✔
Netherlands	✔	✔	✔	✔	✔	✔	✔
Spain	✔	✔	✔	✔		✔	✔
Sweden	✔	✔	✔	✔		✔	
UK	✔	✔	✔	✔	✔	✔	✔
**Oceania**							
Australia	✔	✔	✔	✔	✔	✔	✔
New Zealand	✔	✔	✔	✔		✔	✔

^a^Information about admission type (e.g., urgent, emergent, elective)

^b^Information about discharge disposition (e.g., to home, transfer to another hospital, to long-term care, etc.)

^c^Physician information (medical specialty of physician responsible for care)

^d^Diagnosis timing (e.g., present on admission, developed after admission)

### Country-specific data collection characteristics

When asked to report on other important features of their data collection system, 10 of the 26 countries responded. Half of these were low- and middle-income countries [14] who reported on instances whereby ICD coding was not used, or ICD coding was used in tandem with a different coding guideline. Three of 4 African countries (Mauritius, Nigeria and Tanzania) provided information regarding a misuse of ICD, either in private settings, emergency departments, or remote areas. Indonesia reported use of an amalgamation of ICD-9 and ICD-10, where ICD-10 is used for measuring morbidity, and ICD-9 is used for measuring severity and for billing purposes.

The other half were high-income countries [14] who described their national standards for coding, or a lack thereof, and how these standards contributed to uniformity in coding. Chile, United Kingdom, and Australia provided information on national legislation that mandates coding standards, as well as additional steps taken by coders to meet coding requirements. Although all 26 countries had initially reported having national coding standards, in Sweden, Guatemala, and Nigeria respondents from the same country differed among. Participants from Sweden further clarified that, while national requirements for data collection exist, there are no national standards for clinical documentation, and rather had 21 independent regional health authorities designing coding documentation. Both high- and low-income countries were concerned with coding guidelines (ICD or other) and coding standards that influenced the uniformity and quality of their country’s coded data.

We inquired into whether international countries used electronic abstracting systems in hospitals to help the collection of coded data. Of the 26 countries, 13 used electronic abstracting systems, 11 did not, and 2 countries were unsure. While most countries did not specify the name of the abstracting system they utilized, the majority used locally-developed or regional vendors that were not widely known (*n* = 9), while 4 countries used commonly known vendors such as EPIC® and 3M™.

## Discussion

Lack of comparability in international health data can lead to issues in unnecessary resource use, an increase in reporting errors and omissions, and continuous use of crosswalk and mapping between systems [3]. We conducted an international online questionnaire to better understand the differences in ICD coding practices and hospital data collection systems across countries. Results from 47 participants from 26 countries revealed variances in all aspects of their hospital morbidity data collection systems, with special mention to the disparities in ICD-meta-features: the maximum number of coding fields allowed for diagnosis, the definition of main condition, and diagnosis timing as a mandatory data field to be captured in the hospital morbidity database. Ultimately, the results of the current survey might encourage countries to enhance the quality of their hospital morbidity databases and administrative health data. In particular, these findings offer insights regarding the potential to achieve greater comparability with adoption of the new ICD-11 for Mortality and Morbidity Statistics (ICD-11 MMS).

To our knowledge, this is the first survey inquiring about the number of coding fields for both diagnosis and hospital interventions across countries. Consistent with our results, a similar prior survey had explored ICD use, although they only reported on 9 countries and focused exclusively on the diagnosis coding fields [15]. The effects of limiting the allowable number of diagnosis fields in hospital administrative data had been investigated previously in the literature [15–20]. These studies coincided in their conclusions: there is a decrease in prevalence estimates and accuracy of coding (undercoding) when the number of coding fields is substantially reduced (e.g., truncating original abstracts from 25 to 5 diagnosis fields). When countries have a different number of diagnosis coding fields compared to one another, the clinical complexity of a hospitalized patient is not adequately reflected in their administrative health data and may lead to undercoding of health conditions. The current study reinforced that these differences still exist, and thus the quality and comparability of the data is affected.

Furthermore, it was previously identified that the definition used for describing the “main condition” or the principal diagnosis differ internationally [21], which was also reflected in the results of the current study. However, through the years, there have been changes in the main condition definition used by each country. For example, a country that reported using “reason for admission” in 2014 may have switched to using “main resource use” in our survey, which is the recommended definition by the WHO for the ICD-10 version. The lack of standardization in data collection systems affects the validity and usability of ICD-coded data within and across countries. Data with low validity can affect case selection and inferences made from coded data research, thus resulting in selection bias. More specifically, low validity could lead to issues such as: i) underestimating the disease burden, ii) inaccurate adjustment for severity of illness in assessing for quality and safety, and iii) incorrect calculation of estimated costs generated from disease grouping methodology for hospital payment [21]. Therefore, harmonizing and establishing consistent use of ICD meta-features for morbidity datasets should be a common goal to reduce variations in analyses.

To improve morbidity information in ICD-coded hospital data, the WHO Topic Advisory Group on Quality and Safety (QS-TAG) recommends expanding the number of coding fields to 15–20 (at least) [15], as well as adopting the diagnosis timing flag [22]. The diagnosis timing flag indicates whether conditions were developed in-hospital or prior, and has been incorporated into ICD-11 MMS. Combining these recommendations from the QS-TAG with enhanced education of healthcare providers and coders has also been suggested for optimal capture of clinical information [19, 22, 23]. It is important that the number of data fields and conditions captured is balanced with feasibility of data collection, as coders and coding managers have reported that data quality and quantity of the data collected must be balanced with meeting timelines and quotas set by their jurisdictions [24, 25].

Beyond the variations in ICD coding and data collection across countries, there are other factors that prevent adequate comparative analysis of international hospital data [15]. Such factors include documentation quality within medical charts, hospital payment mechanisms (financial incentives), coding guidelines, as well as ICD versions and modifications [3, 19, 26, 27]. As an example, the quality of the output (ICD codes) depends on the quality of the input (information documented in the medical chart). Missing data in the medical chart would decrease the prevalence of certain diseases and result in undercoding [26]. In parallel, reimbursement methods in some hospitals are based on diagnosis-related groups. Further, financial incentives might lead physicians to choose one diagnosis over the other, thus resulting in subsequent over- or undercoding of that condition [27]. By standardizing the features used for coding, countries can reduce within- and across-country differences in data collection processes, and ultimately increase the accuracy of disease burden estimates.

Further adding to the complexity of international coded data comparability are the modifications that various countries have adopted to meet their specific healthcare needs and contexts. For ICD-10, these countries include Australia (ICD-10-AM), Canada (ICD-10-CA), Germany (ICD-10-GM), and Korea (ICD-10-KM). Modified versions include an increased number of codes, and code-specific changes at the more granular levels (4th, 5th and 6th digit-levels). Certain categories of codes exist in one country but may not exist in another, limiting the ability to compare certain clinical contexts. For example, the ICD-10-GM has a chapter specific to behavioral and mental disorders (Chapter 5), whereas the ICD-10-CA does not, thus limiting the comparability of these disorders between the two countries [28, 29]. Issues with comparability will endure when countries do not adopt ICD systems simultaneously, or only adopt portions of it [3]. For example, in the current study, we found that some countries adopted ICD-10 for morbidity coding, but continued to use ICD-9 for coding severity and billing purposes. Finally, it is infeasible for some developing countries to adopt ICD, as it requires complex coding processes (e.g., advanced electronic infrastructure). To accommodate for hardware or software limitations, developing countries have created simplified versions of ICD, and with multiple versions in use, [5], international comparability is compromised.

The current study has limitations. The response rate was suboptimal (26 of the 117 countries currently using ICD), which is an inherent disadvantage of online surveys. To mitigate this, snowball sampling was used to reach as broad an audience as possible, followed up with reminder emails. There is also the potential for selection bias, particularly non-response bias, by which certain types of survey respondents (those countries who did not respond) are under-represented. As such, it is possible that the full extent of ICD and hospital morbidity data features was not captured from many countries. However, we did observe that our responses reached a certain level of saturation, and that our conclusion will remain that variability in data collection features exist. Despite the small number of responses, there were survey participants representing every continent, thereby gaining a deeper understanding of ICD and hospital morbidity data collection features worldwide. Furthermore, the survey results could incentivize countries to participate in future research, so that they may be able to understand how their data compares to other countries on a global scale. Another difficulty emerged during data analysis when grouping responses geographically, as sometimes there was more than one participant from the same country and their replies were discordant. When this happened, all answers were included and disclosed in the results for transparency. Conflicting information within countries may be explained by the lack of standards both at national and international levels. Further, the current questionnaire did not inquire how the hospital morbidity database was used in the participants’ country (e.g., for research, health system administration, etc.), thus we do not know the extent to which usability was impacted by data quality and the differences in data collection features. Lastly, the questions posed in the survey may have had a general quality to them, as they were not made to be granular, knowing some variation existed. General questions may have sacrificed some specificity for the characterization of data features, in order to offer a universally-interpretable survey. We also offered open-ended questions as options for countries to provide details about their unique data collection characteristics.

## Conclusion

These survey data demonstrated the current status of ICD and hospital morbidity data collection features internationally. Results will be reported to WHO to illustrate the landscape of differences in ICD meta-features, and prepare for the adoption of ICD-11 MMS. Thus, the current survey could assist in enhancing data comparability twofold: 1) by bringing awareness to heterogeneity in data collection across countries, in an effort to better data quality for research and surveillance purposes, and 2) by informing stakeholders and decision-makers of the need to improve and support standardization of ICD data in the future.

## Supplementary Information

**Additional file 1.** Survey Questions and Answers (English version).

## Data Availability

The datasets analyzed during the current study are available from the corresponding author on reasonable request.

## Abbreviations

ICD: International Classification of Diseases
WHO: World Health Organization
ICD-9: International Classification of Diseases, 9th version
ICD-10: International Classification of Diseases, 10th version
ICD-11 MMS: International Classification of Diseases, 11th version for Mortality and Morbidity Statistics
WHO-FIC: World Health Organization Family of International Classifications
PAHO: Pan American Health Organization
IFHIMA: International Federation of Health Information Management Association
RELACSIS: Red Latinoamericana y del Caribe para el fortalecimiento de los Sistemas de Salud (*transl.* the Latin American and Caribbean Network for the enhancement of Health Systems)
OECD: Organisation for Economic Co-operation and Development
QS-TAG: WHO Topic Advisory Group on Quality and Safety
