# Supplementary material for: Exploring the differences in ICD and hospital morbidity data collection features across countries: an international survey
Source: BMC Health Serv Res. 2021 Apr 7;21:308. doi: 10.1186/s12913-021-06302-w (PMC8025494; doi:10.1186/s12913-021-06302-w)
Supplement: Supplementary file 1 — Additional file 1. Survey Questions and Answers (English version). [file 12913_2021_6302_MOESM1_ESM.pdf]

Additional file 1. Survey Questions and Answers (English version)

1. In what country do you currently live? Specifically, what country's data will you be describing?
2. Does your country collect hospital morbidity data? (Morbidity - the state of being diseased or unhealthy within a population)
   - ○ Yes ○ No ○ I don't know
3. Do you currently use ICD (As of July 1, 2017) for coding hospital morbidity data?
   - ○ Yes ○ No ○ I don't know
   - ○ If No, then how do you collect hospital morbidity data?
4. Are there national standards for ICD-coded data collection in your country?
   - ○ Yes ○ No ○ I don't know
   - ○ If No, at what level are standards set for data collection using ICD codes (regional, local,...)?
5. What is the maximum allowable for diagnosis coding fields in your hospital morbidity data?
   - ○ 1-6 ○ 31 or more (limited)
   - ○ 7-15 ○ Unlimited
   - ○ 16-30 ○ I don't know
6. Which types of data fields are mandatory or required in your hospital morbidity database?
   - ○ Patient demographics
   - ○ Information about admission type (e.g., urgent, emergent, elective)
   - ○ Information about discharge disposition (e.g., to home, transfer to another hospital, to long-term care, etc.)
   - ○ Admission unit (e.g., cardiology, dermatology, rheumatology, ...)
   - ○ Physician information (medical specialty of physician responsible for care)
   - ○ Diagnoses
   - ○ Diagnosis timing (e.g., present on admission, developed after admission)
   - ○ Not applicable (Please select this option if there are no mandatory or required fields)
7. Are hospital interventions (e.g., surgery, diagnostic tests,...) coded in your hospital morbidity database?
   - ○ Yes ○ No ○ I don't know
   - ○ If Yes, what coding system is used for coding hospital interventions?
8. What is the maximum allowable for hospital interventions coding fields in your hospital morbidity data?
   - ○ 1-6 ○ 31 or more (limited)
   - ○ 7-15 ○ Unlimited
   - ○ 16-30 ○ I don't know
9. What is the definition of "main condition" in your country?
   - ○ Reason for admission (The condition established after study to be chiefly responsible for causing the admission of the patient to the hospital for care)
   - ○ Resource use (Most responsible diagnosis for the patient's stay in hospital. If there is more of such condition, the one held most responsible for the greatest portion of the length of stay or greatest use of resources)
   - ○ Other (please specify)
10. Are there any other important features of the data collection system in your country that you would like to share?
11. Do you use an electronic abstracting system(s) in hospitals?
    - ○ Yes ○ No ○ I don't know
    - ○ If Yes, which electronic abstracting systems do you use (vendor name, the name of the system)?
